# Adjuvant hormonal therapy for stage I endometrial cancer

**DOI:** 10.3747/co.v15i3.204

**Published:** 2008-06

**Authors:** L. Gien, J. Kwon, T.K. Oliver, M. Fung-Kee-Fung

**Affiliations:** * Division of Gynecologic Oncology, University Health Network, Princess Margaret Hospital, Toronto, ON; † Division of Gynecologic Oncology, M.D. Anderson Cancer Center, Houston, TX, U.S.A; ‡ Clinical Epidemiology and Biostatistics, McMaster University, Hamilton, ON; § Division of Gynecologic Oncology, The Ottawa Hospital Regional Cancer Centre, Ottawa, ON

**Keywords:** Adjuvant hormonal therapy, stage i endometrial cancer, early-stage endometrial cancer

## Abstract

**Question:**

What is the role of hormonal therapy as adjuvant therapy in patients with stage i endometrial cancer?

**Perspectives:**

There is little consensus on the role of adjuvant treatment for patients with stage i endometrial cancer. Although the use of hormonal therapy has been established in advanced disease, less agreement has emerged concerning the benefits of adjuvant hormonal therapy for patients with early-stage disease. The objective of the present evidence series was to review the existing literature on the role of hormonal therapy as adjuvant therapy in patients with stage i endometrial cancer.

**Outcomes:**

Reports were sought that included at least one of the following outcomes: overall survival, disease-free survival, recurrence (local, or distant, or both), adverse effects, and quality of life. Because of the potential for long-term adverse effects with adjuvant hormonal treatment in this patient population, especially with regard to thromboembolic or cardiovascular events, the rates of non-cancer-related death were also of interest.

**Methodology:**

The medline, embase, and Cochrane Library databases were systematically searched for randomized controlled trials, practice guidelines, systematic reviews, and meta-analyses. The resulting evidence informed the development of the clinical practice guideline. The systematic review with meta-analyses and practice guideline were approved by the Report Approval Panel of the Program in Evidence-Based Care, and by the Gynecology Cancer Disease Site Group (dsg).

**Results:**

Nine randomized trials and one published meta-analysis comparing adjuvant hormonal therapy with no adjuvant therapy in women with stage i endometrial cancer constituted the evidence base. One trial reported a statistically significant survival benefit with adjuvant progestogen as compared with no further treatment (97% vs. 69%, *p* < 0.001). In that trial, the treatment group had a higher number of patients with less myometrial invasion, and a lower number of patients with advanced-stage disease. These differences in baseline characteristics between the randomized groups were considered to be clinically important. In addition, the results of that trial were not consistent with those of other trials, and the trial was a source of statistical heterogeneity when data were pooled across trials.

In two of the nine randomized trials, statistically significant recurrence-free benefits were detected with adjuvant hormonal therapy as compared with no further therapy. In one trial, the difference between the rates of recurrence was 16%; however, the methodologic concerns related to that that trial limited its relevance. In the other trial, the difference between the rates of recurrence was 5%. In that trial, patients were at a high risk of recurrence. None of the remaining seven randomized trials reported any significant difference in recurrence rates between treatment groups.

The meta-analysis identified in the literature detected no statistically significant recurrence-free or overall survival benefit associated with adjuvant hormonal therapy as compared with no adjuvant therapy [odds ratio (or): 1.05; 95% confidence interval (ci): 0.88 to 1.24). Those results are consistent with the results of the meta-analysis in the present report, which included an additional two trials (or: 1.10; 95% ci: 0.91 to 1.34).

**Practice Guideline:**

**Target Population**

This clinical recommendation applies to women with newly diagnosed stage i endometrial cancer.

**Recommendation**

The available evidence does not demonstrate any benefit for adjuvant hormonal therapy. The use of hormonal therapy is not recommended as adjuvant treatment for patients with stage i endometrial cancer.

## 1. QUESTION

What is the role of adjuvant hormonal therapy in patients with stage i endometrial cancer?

## 2. CHOICE OF TOPIC AND RATIONALE

Endometrial cancer is the most common gynecologic cancer in Canada 1. Each year, approximately 3900 women are diagnosed with endometrial cancer in Canada, about 1550 of whom reside in the province of Ontario 1. Approximately 75% of patients present with stage i disease, which is confined to the uterus^2^. Primary surgical treatment for patients with stage i endometrial cancer typically consists either of a total abdominal hysterectomy with bilateral salpingo-oophorectomy, or complete surgical staging, which involves abdominal hysterectomy, bilateral salpingo-oophorectomy, dissection or sampling of pelvic or para-aortic nodes (or both), peritoneal cytology, omentectomy, and peritoneal biopsies 3.

There is little consensus on the role of adjuvant treatment for patients with stage i endometrial cancer. Currently, there is evidence to support the role of adjuvant pelvic radiotherapy in stage i endometrial cancer to reduce risk of recurrence for higher-risk patients (stage ic, grade 3) and perhaps for patients at intermediate risk (stage ic, grades 1 or 2; or stage ia or ib, grade 3), but not for patients at lower risk (stage ia or ib, grades 1 or 2) 4. Although the use of hormonal therapy has been established in advanced disease 5, less agreement has emerged on the role of adjuvant hormonal therapy in early-stage disease.

The objective of the present evidence series was to review the existing literature on the role of hormonal therapy as adjuvant therapy in patients with stage i endometrial cancer.

## 3. METHODS

### 3.1 Literature Search Strategy

A literature search was conducted using medline (ovid: 1966 through January 2007), embase (ovid: 1988 through January 2007), the Cochrane Library database (ovid: Issue 1, 2007), the Physician Data Query database, the Canadian Medical Association InfoBase, and the National Guideline Clearinghouse. In addition, abstracts published in the proceedings of the meetings of the American Society of Clinical Oncology (1997–2006) and the European Society for Medical Oncology (2002–2006) were searched for evidence relevant to this report. Reference lists of related papers and recent review articles were also scanned for additional citations.

The literature search of the electronic databases combined disease-specific terms (endometrial neoplasms/ or endomet:.ti. and cancer.ti. or neoplasms/ or carcinoma:.ti. or adenocarcinoma:.ti.) with treatment-specific terms (antineoplastic agents, hormonal/) for the following study designs: randomized controlled trials (rcts), practice guidelines, systematic reviews, and meta-analyses.

### 3.2 Study Selection Criteria

Articles were selected for inclusion in the systematic review of the evidence if they randomized patients with stage i endometrial cancer either to adjuvant hormonal therapy or to no adjuvant treatment or other forms of hormonal therapy. To encompass trials in which most patients had early-stage disease, an *a priori* decision was made to include trials if at least 60% of the patients reported had stage i disease or if results were reported separately for patients with stage i disease. The report had to include at least one of the following outcomes: overall survival, disease-free survival, recurrence (local, or distant, or both), adverse effects, or quality of life. Because of the potential for long-term adverse effects with adjuvant hormonal treatment in this patient population, especially with regard to thromboembolic or cardiovascular events, the rates of non-cancer-related death were also of interest. It was determined *a priori* that the literature search would be expanded to include other study designs if the initial search failed to identify sufficient evidence to inform the systematic review.

Practice guidelines, meta-analyses, or systematic reviews explicitly based on evidence related to the guideline question were also eligible for inclusion in the systematic review.

Articles were excluded from the systematic review of the evidence if they were case reports, letters, editorials, or papers published in a language other than English.

### 3.3 Synthesizing the Evidence

Combining results across trials provides added power for detecting the efficacy of a treatment and improves the reliability or confidence of the point estimate. Ideally, data are pooled using hazard ratios (hrs); however, if that method is not possible given the level of the data reporting, meta-analyses using point-in-time estimates are conducted.

For the meta-analysis reported here, data were analyzed using the Review Manager 4.2.10 statistical package (The Nordic Cochrane Centre, Copenhagen, Denmark), obtained through the Cochrane Collaboration (www.cochrane.org). Results are expressed as the pooled hr or the odds ratio (or) with 95% confidence interval (ci), in which a value less than 1.0 favours the experimental treatment and a value greater than 1.0 favours the control.

As part of combining data in a meta-analysis, an assessment of heterogeneity is performed. Clinical heterogeneity is assessed by determining whether the populations, interventions, and outcomes are sufficiently similar to pool data. Statistical heterogeneity was assessed by *Q* test, with a value of *p* < 0.10 selected as the level at which heterogeneity is deemed to be present. The *I*^2^ statistic quantifies how much heterogeneity can be attributed to chance or to a real effect. If substantial heterogeneity is present, possible clinical and methodologic reasons are qualitatively explored. The random effects model is typically chosen over the fixed effects model as the more conservative estimate of effect.

## 4. RESULTS

### 4.1 Literature Search Results

Nine s 6–14 and one published meta-analysis 15 rct on adjuvant hormonal therapy for patients with stage i endometrial cancer met the specified criteria and were deemed eligible for inclusion in the systematic review of the evidence. Because the number of rcts identified was sufficient, the search was not expanded to include other study designs.

In the nine trials identified, patients were randomized to either adjuvant progestogen therapy or to a control group with no adjuvant therapy 6–14. In seven trials, patients in the control group received no further treatment 6–12; in two trials, patients in the control group received a placebo control. One trial also included a third group of patients randomized to receive tamoxifen 6.

### 4.2 Study Quality

Only two of the nine randomized trials specifically described the randomization process 6,11; however, an additional two trials 9,14 were multicentric investigations that reported centralized randomization procedures. Thus, although randomization and allocation concealment techniques were not specifically reported, it can be inferred that adequate randomization occurred in the latter two trials. Each of the trials reported a comparison of baseline patient characteristics and important prognostic variables that could potentially influence outcome, such as depth of myometrial invasion and histologic grade. In seven trials 6–7,9–13, baseline characteristics of the patients in the treatment and control groups were comparable, demonstrating an adequate randomization process. In the trial by Urbanski *et al.* 8, the progestogen group had noticeably more patients with favourable characteristics in terms of stage and depth of myometrial invasion, a clinically relevant difference between groups. Conversely, in the trial by Lewis *et al.* 14, the placebo group had more patients with favourable characteristics with regard to depth of myometrial invasion.

All of the trials except the trial by Quinn 7 maintained their comparison groups. In the Quinn trial, if recurrence was observed in the control group, the patients were offered adjuvant hormonal therapy and crossed over to the treatment group.

Overall survival 6–11,13–14 or recurrence-free survival 12 were described or inferred as the primary outcomes of interest upon which power calculations were based. Only two trials specifically reported their power calculation 7,11, but omission of that information may be a reflection of how reporting standards for clinical trial methodology have changed over time. Five trials randomized more than 200 patients per arm 7,9–11,14, two trials 6,8 randomized fewer than 135 patients per arm, and two trials 12,13 randomized fewer than 30 patients per arm.

An intent-to-treat analysis was reported in five of the nine trials 7–9,11,13 and not reported in the remaining four trials 6,10,12,14. Intent to treat is a more conservative approach to data analysis whereby all patients are analyzed according to their original randomization assignment regardless of factors such as drop-out, non-compliance with treatment, or crossover. Seven studies had a follow up rate of >80% 6,8,10–14, one study had a follow-up of 75% 9, and one study did not report follow-up data 7. Where reported, overall survival was measured at or beyond the point of median follow-up.

### 4.3 Trial Characteristics

To be eligible to participate in the trials, patients required a histologic diagnosis of endometrial adenocarcinoma and, with the exception of one trial 14, first had to undergo surgery, which included at least a total abdominal hysterectomy and bilateral salpingo-oophorectomy, followed by adjuvant radiation therapy if indicated according to pathology criteria. In the trial by Lewis *et al.* 14, patients were randomized into two groups, one offering preoperative radiation therapy and surgery, and one offering surgery alone. (Current practice no longer uses preoperative radiation therapy.) The progestogen agents in the adjuvant hormonal therapy groups included medroxyprogesterone acetate 6,7,9,11,14, hydroxyprogesterone caproate 8,10,13, and gestonorone caproate 12. The doses of hormonal therapy were relatively equivalent in all of the trials, even when compared across formulations.

In four trials 9,12–14, only patients with stage s disease (confined to the uterine corpus) were eligible to participate; in the remaining five trials 6–8,10,11, patients with more-advanced stages of endometrial cancer were also eligible. In the latter trials, at least 60% of patients were diagnosed with stage s disease 6–8,10,11. The trial reported by Quinn had specific inclusion criteria for patients with histologically high-risk endometrial cancer in addition to stage s disease 7. Furthermore, that trial performed a subgroup analysis of the patients after removing 117 women considered ineligible after pathology review. In three other trials, subgroup analyses of high-risk endometrial cancer were performed 6,10,12. In those three trials, the subgroup analyses were not decided *a priori,* and they were therefore likely not to have adequate power for significant results. In the present review, data on subgroup analyses are not reported.

Protocols for changes in treatment were specified in three of the nine randomized trials, with patients who experienced a recurrence in the control group being able to cross over to the hormonal treatment group s or with patients who experienced side effects having the option to stop treatment 9,11. In the trials in which patients had the option to stop treatment because of side effects, patients who stopped treatment were still analyzed according to the intent-to-treat approach 9,11.

The trials were conducted in Germany 6,Australia 7, Poland 8, Italy 9,12, Norway 10, the United Kingdom 11, and the United States 13,14.

### 4.4 Outcomes

Does hormonal therapy used as adjuvant treatment for early-stage endometrial cancer improve recurrence and survival outcomes for patients, and is overall quality of life affected? Is hormonal therapy more likely to be useful for well-differentiated tumours than for poorly-differentiated tumours?

The sections that follow outline key findings regarding recurrence rates, survival, and adverse events, including non-cancer-related deaths.

No identified trial answered the questions regarding quality of life or tumour differentiation, nor did they report compliance with treatment. It should also be noted that reporting conventions have changed over time, and because the identified trials span a 30-year period, some data considered standard by today’s conventions are missing in the reporting of the trials (for example, reporting of hrs, common-point-in-time estimates, adverse events, compliance with treatment, and so on). Data from the trials were extracted as available. Table I summarizes the recurrence and survival results of the nine trials.

#### 4.4.1 Survival

Of the nine randomized trials, one trial detected a statistically significant difference in overall survival between the treatment group, who received hydroxyprogesterone caproate, and the control group, who received no further treatment 8. In that trial (by Urbanski *et al.*), the overall survival rate at 5 years was 97% in the treatment group as compared with 69% in the control group. Although 70% of the patients had stage i disease, the foregoing results were based on all 205 patients with stages i–iii endometrial cancer. A criticism of the trial is that the prognostic variables in the two groups were not equally distributed at baseline: the treatment group had a higher number of patients with less myometrial invasion, and a lower number of patients with advanced-stage disease. These differences in baseline characteristics between the randomized groups were considered to be clinically important.

The remaining eight trials did not find any statistically significant differences between the treatment and control groups 6,7,9–14. The overall survival results from the trial reported by Quinn 7 may have been tempered by the crossover of 64 of the 107 control patients to hormonal therapy at recurrence; however, outside of subgroup analyses, the effect of crossover on survival is unknown.

The data were sufficient to pool the total number of deaths in the treatment and the control groups across all trials 6–14; however, because the data reporting was variable, it was not possible to conduct meta-analyses using hrs, and identical points in time could not be captured. The total numbers of deaths were pooled across different time points as an alternative method of pooling data. In one trial in which data were not directly reported, data were estimated from information reported in the trial using the intent-to-treat population as the denominator 12.

**FIGURE 1:**
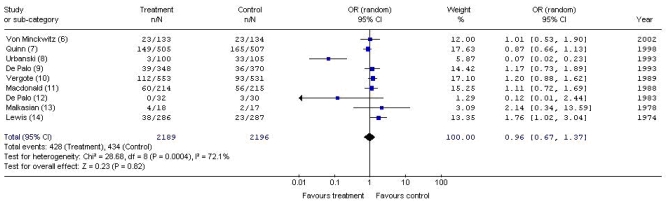
Meta-analysis of deaths with adjuvant hormonal therapy versus control (9 trials). or = odds ratio; ci = confidence interval.

**FIGURE 2:**
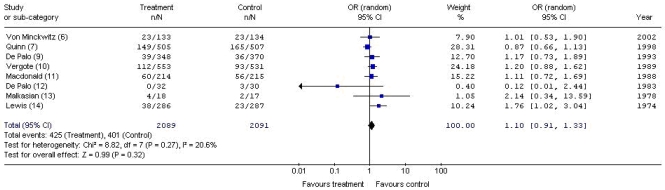
Meta-analysis of deaths with adjuvant hormonal therapy versus control (8 trials). or = odds ratio; ci = confidence interval.

As seen in Figure 1, meta-analysis demonstrated no statistically significant difference in overall survival between patients who received an adjuvant hormonal therapy and patients in the control groups (or: 0.96; 95% ci: 0.67 to 1.37). The data were statistically heterogeneous (*p* = 0.0004, *I*^2^ = 72.1%), and sensitivity analysis determined that the source of the statistical heterogeneity was the trial by Urbanski *et al.* 8. Given that the Urbanski *et al.* trial was the source of the statistical heterogeneity and that the unequal distribution of prognostic variables at baseline in that trial was clinically relevant, that trial was removed from the pooled analysis. As seen in Figure 2, when the Urbanski *et al.* 8 trial was removed, the heterogeneity of the meta-analysis was no longer statistically significant (*p* = 0.27, *I* 2 = 20.6%), and the difference in overall survival remained nonsignificant between groups (or: 1.10; 95% ci: 0.91 to 1.34).

#### 4.4.2 Recurrence

Recurrence rates were reported in six of the nine randomized trials 6–10,12. Of those six trials, two reported a statistically significant difference in recurrence rates between treatment groups 7,8. In the trial by Quinn 7, in which all patients were at a high risk of recurrence, 21% of the control group as compared with 16% of the hormonal treatment group experienced a relapse (*p* < 0.05). In the trial by Urbanski *et al.* 8, 23% of patients in the control group as compared with 7% of the patients in the hormonal therapy group recurred (*p* < 0.001). The remaining four trials showed no statistically significant difference in recurrence rates between the treatment and control groups 6,9,10,12.

Six of the nine trials provided sufficient information to pool the total number of recurrences in both the treatment and control groups using point-in-time estimates 6–10,12. The reporting of data was again variable across the randomized trials, and thus the total numbers of recurrences were pooled across various time points. Data were estimated from information reported in one trial using the intent-to-treat population as the denominator 12.

**FIGURE 3:**
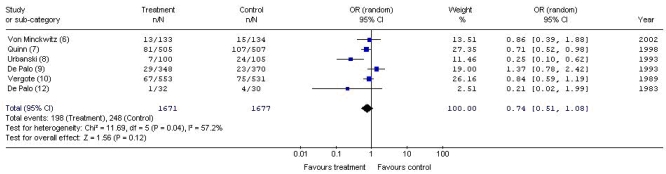
Meta-analysis of recurrences with adjuvant hormonal therapy versus control (6 trials). or = odds ratio; ci = confidence interval.

**FIGURE 4:**
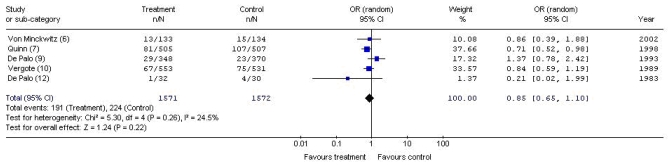
Meta-analysis of recurrences with adjuvant hormonal therapy versus control (5 trials). or = odds ratio; ci = confidence interval.

As seen in Figure 3, although fewer recurrences were associated with adjuvant progestogens, no statistically significant differences in recurrence rate were detected between patient groups (or: 0.74; 95% ci: 0.51 to 1.08) and statistically significant heterogeneity was detected (*p* = 0.04, *I*^2^ = 57.2%). The source of the statistical heterogeneity was once again deemed to originate in the trial by Urbanski *et al.* 8. As seen in Figure 4, when that trial was removed from the pooled analysis, the remaining five trials 6,7,9,10,12 were statistically homogeneous (*p* = 0.26, *I*^2^ = 24.5%), and the difference in recurrence remained nonsignificant between groups (or: 0.85; 95% ci: 0.65 to 1.10).

### 4.5 Adverse Events

Table II summarizes the adverse effects reported across the nine randomized trials 6–14. Five trials reported data on major harmful side effects or deaths unrelated to the malignancy, and three trials reported on minor symptomatic side effects or withdrawals because of toxicity 6,9,11. The most common minor side effects included weight gain, peripheral edema, and nausea 6,9. One trial reported an incidence of overall minor side effects of 53% in the progestin group and 16% in the control group, but did not indicate whether the difference was statistically significant 6. Another trial reported a minor side effect rate of 12%, but did not calculate side effects for patients in the control group 9. Macdonald *et al.* reported that 4% of patients in the hormonal therapy group developed hypertension, which disappeared after the drug was stopped 11. The drop-out rate attributable to toxicity ranged from 5% to 19% in the three trials that reported data on that outcome 6,9,11.

Serious side effects included thromboembolic events such as deep vein thrombosis, pulmonary embolus, and stroke, or cardiovascular disease such as myocardial infarction and deterioration of congestive heart failure. In the trials that reported these events, there were no statistically significant differences reported between the treatment and control groups 6,7,9–11. One trial 6 indicated a serious side effect rate of 6% in the progestogen group as compared with 2% in the control group (*p* value not reported), and another trial 10 reported higher rates of death from cardiovascular disease in the first 2 years in the progestogen group than in the control group (5% vs. 3%, *p* = 0.07). Additionally, deaths that were not attributable to malignancy were related mainly to cardiovascular or thromboembolic causes. Only one trial^10^ showed a statistically significant difference in deaths unrelated to malignancy (9% of patients in the hormonal group vs. 6% of patients in the control group, *p* = 0.04). In the trial by Urbanski *et al.* 8, the only trial to report an overall survival difference, 11 patient deaths unrelated to malignancy occurred in the control arm, and none occurred in the treatment arm (*p* value not reported). The remaining trials that reported deaths unrelated to malignancy did not detect any statistically significant difference between the treatment and control groups 6,7,9,11.

### 4.6 Quality of Life

None of the studies reported data on quality of life.

### 4.7 Meta-Analysis Identified by the Literature Search

Martin–Hirsch *et al.* 15 conducted a meta-analysis of published data comparing adjuvant progestin therapy to no adjuvant therapy in endometrial cancer. The authors identified seven 7–11,13,14 of the nine trials 6–14 included in the present systematic review of the evidence. These authors also excluded the trial by Urbanski *et al.* 8 on the basis of clinical and statistical heterogeneity, and reported no significant difference in overall survival between patients who received progestin therapy and those who received no adjuvant treatment (or: 1.05; 95% ci: 0.88 to 1.24; *p* = 0.6). They also identified three trials that reported recurrence rates 7,9,10. Again, with the removal of the trial by Urbanski *et al.* 8, a marginal reduction in recurrence rate was detected among women receiving progestin therapy as compared with women receiving no adjuvant therapy (or: 0.81; 95% ci: 0.65 to 1.01; *p* = 0.06); however, the rate was not statistically significant. Meanwhile, the authors reported that the rate of non-cancer-related deaths was significantly higher in the progestin group (or: 1.33; 95% ci: 1.02 to 1.73), presumably because of the adverse cardiovascular effects of progestin treatment.

The meta-analysis in the present review of the evidence included two additional trials, the trials by von Minckwitz 6 and De Palo 12. Overall, aside from the additional trials, the results of the two meta-analyses were highly comparable.

## 5. CONCLUSIONS

Nine randomized trials and one meta-analysis of published data provide the evidence base for assessing the role of adjuvant hormonal therapy in women with stage i endometrial cancer. Several factors limit the interpretability of the results, but the greatest limiting factor is that the trials, which span a 30-year period, generally have inconsistent reporting throughout. The inconsistencies limit the quality assessment of internal validity related to patient and study characteristics and to outcomes. No quality-of-life data, little data on adverse events or treatment compliance, and limited data on recurrence and survival outcomes, especially in regard to hrs and time-to-event estimates, were reported. There were also differences in patient populations, unexpected findings that were not consistent with the results of similar randomized trials, and notable discrepancies between patients at baseline despite the randomization process. These limitations affected the external validity of the trials; however, these trials provide the only randomized data that inform the role of adjuvant hormonal therapy in this patient population.

Despite the limitations, the evidence was consistent in the direction of effect: adjuvant hormonal therapy does not confer a survival advantage in patients with stage i endometrial cancer. Eight of the nine randomized trials failed to detect any difference in survival between the treatment group and the control group. The remaining trial did demonstrate a survival difference, but the quality of that trial is subject to criticism because of important differences in baseline characteristics between the patient groups. Moreover, the trial results were not consistent with the results of the other identified rcts. The magnitude of the effect is highly unexpected when reviewed in comparison with the results of the other similar trials reported. Also, in two meta-analyses, no survival advantages were detected with adjuvant hormonal therapy.

In seven of the nine randomized trials, recurrence rates were not significantly different between patients in the adjuvant hormonal groups and patients in the control groups. Of the two trials that detected lower recurrence rates in patients in the progestin group, the trial by Urbanski *et al.* 9 reported more favourable baseline characteristics for patients in the treatment group, and the trial by Quinn 7 reported data on patients at high risk of recurrence. Although the meta-analysis in the present review and the previously published meta-analysis showed a marginal reduction in recurrence rate with adjuvant therapy, that reduction was not statistically significant at the 0.05 level.

Finally, although not consistently reported, the rates of adverse events among patients in the hormonal groups were generally higher than those among patients in the control groups. Minor side effects were reported more often in the treatment groups, although tests of statistical significance were not performed. The rate of non-cancer-related death was shown to be higher with progestogen in one randomized trial, mainly because of cardiovascular or thromboembolic events (*p* = 0.04). In contrast, Urbanski *et al.* 8 reported a 10% non-cancer-related death rate in the control group and a 0% rate in the treatment group. This unexpected finding not seen in the other randomized trials. The meta-analysis by Martin–Hirsch *et al.* 15 did not demonstrate a statistically significant difference in non-cancer-related death.

Unfortunately, the data reported in the randomized trials were insufficient to stratify patients with stage i disease by high as compared with low risk of recurrence. Whether the subgroups of patients who were at a higher risk of recurrence (stage ic, grade 3) would or would not have benefited from adjuvant hormonal therapy cannot be fully answered by the available data. Interpretation is further confounded by the fact that five of the nine randomized trials included between 10% and 35% of patients with greater than stage i disease. Further investigation by risk of recurrence should be the focus of future research.

Given the lack of an overall survival benefit, the marginal decrease in recurrence rate (seen mainly in patients at higher risk of recurrence), and the need for treatment regimens that can span years with possible increases in adverse events, the evidence is currently insufficient to support the use of hormonal therapy as adjuvant treatment for patients with early-stage endometrial cancer.

## 6. FUTURE RESEARCH

The randomized trials completed to date have studied the use of specific types of progestogens in early endometrial cancers, and one trial included tamoxifen as a comparison group 6. Future trials can potentially include other anti-estrogenics, selective estrogen receptor modulators, aromatase inhibitors, and estrogen receptor downregulators in homogenous patient populations.

## 7. CONFLICT OF INTEREST

Members of the Gynecology Cancer dsg were asked to disclose potential conflicts of interest. No conflicts were reported.

**TABLE I:** Randomized controlled trials of adjuvant hormonal therapy in early-stage endometrial cancer

Study	Patients (n)	Treatment groups	Duration	Stage i disease (%)	Median follow-up [months (range)]	Point in time	a Outcomes Recurrence (%)	Overall survival (%)
von Minckwitz *et al.,* 2002 6	134	None	—	82	56	4.5 Years	11	67
	133	Oral mpa 500 mg daily	2 Years	83	(3–199)		10	66
	121	Oral tamoxifen 30 mg daily	2 Years	84			8	70
Quinn, 1998 7	507	None	—	80	65	5 Years	21	72
	505	Oral mpa 200 mg twice daily	≥3 Years	80	(36–120)		16	75
							(*p<*0.05)	
Urbanski *et al.,* 1993 8	105	None	—	65	nr	5 Years	23	69
	100	Intramuscular hpc 500 mg every 2 weeks b	1 Year	78	(nr)		7	97
							(*p<*0.001)	(*p<*0.001)
De Palo *et al.,* 1993 9	370	None	—	100	84	7 Years	6	90
	348	Oral mpa 100 mg twice daily	1 Year	100	(nr)		8	89
Vergote *et al.*, 1989 10	531	None	—	89	72	5 Years	14	82
	553	Intramuscular hpc 1000 mg twice daily c	1 Year	90	(42–132)		12	80
Macdonald *et al.,* 1988 11	215	None	—	72	>60	nr	nr	74
	214	Oral mpa 100 mg daily d	≥5 Years	68	(12–120)		nr	72
De Palo *et al.,* 1983 12	30	None	—	100	nr	5 Years	15	96 e
	32	Intramuscular gestonorone 200 mg weekly	≥1 Year	100	(nr)		4	85 e
Malkasian and Bures 1978 13	17	Intramuscular placebo	14 Weeks	100	60	5 Years	nr	88
	18	Intramuscular hpc 500 mg weekly	14 Weeks	100	(nr)		nr	78
Lewis *et al.,* 1974 14	287	Intramuscular placebo	14 Weeks	100	48	4 Years	nr	92
	285	Intramuscular mpa 500 mg weekly	14 Weeks	100	(nr)		nr	87

a Unless specified, results based on all subjects in the trial, not just stage i patients.

b Some patients received oral doses (numbers not reported).

c Initially, patients in the treatment arm received a loading dose of 5000 mg in the course of 5 days.

d Initial dose for the first year was 100 mg orally three times daily.

e Reviewer’s calculation.

nr = not reported; mpa = medroxyprogesterone acetate; hpc = hydroxyprogesterone caproate.

**TABLE II:** Adverse events of adjuvant hormonal therapy in early endometrial cancer

Study	Patients (n)	Treatment groups	Adverse Minor (%)	Serious events (%)	Withdrawal because of toxicity (%)	Second primary malignancy (%)	Deaths not related to cancer (%)
von Minckwitz *et al.,* 2002 6	134	None	16	2	—	6	9
	133	Progestogen	53	6	19	7	10
	121	Tamoxifen	34	3	3	2	6
Quinn, 1998 7	507	None	nr	4	—	<1	13
	505	Progestogen	nr	5	nr	2	15
Urbanski *et al.,* 1993 8	105	None	nr	nr	—	nr	10
	100	Progestogen	nr	nr	nr	nr	0
De Palo *et al.,* 1993 9	370	None	nr	3	—	3	5
	348	Progestogen	12	2	5	1	4
Vergote *et al.*, 1989 10	531	None	nr	3	—	<1	6
	553	Progestogen	nr	5	nr	1	9 (*p=*0.04)
Macdonald *et al.,* 1988 11	215	None	nr	4	—	nr	11
	214	Progestogen	4^a^	3	11	nr	10
De Palo *et al.,* 1983 12	30	None	nr	nr	—	3	nr
	32	Progestogen	nr	nr	nr	3	nr
Malkasian and Bures 1978 13	17	Placebo	nr	nr	nr	nr	6
	18	Progestogen	nr	nr	nr	nr	11
Lewis *et al.,* 1974 14	287	Placebo	nr	nr	nr	nr	nr
	285	Progestogen	nr	nr	nr	nr	nr

a The only minor adverse event reported was hypertension.

No *p* values were reported, except where indicated. nr = not reported.
